# *KANSL1* gene disruption associated with the full clinical spectrum of 17q21.31 microdeletion syndrome

**DOI:** 10.1186/s12881-015-0211-0

**Published:** 2015-08-22

**Authors:** María Moreno-Igoa, Blanca Hernández-Charro, Amaya Bengoa-Alonso, Aranzazu Pérez-Juana-del-Casal, Carlos Romero-Ibarra, Beatriz Nieva-Echebarria, María Antonia Ramos-Arroyo

**Affiliations:** Medical Genetics Department, Complejo Hospitalario de Navarra, IdiSNA, Navarra Institute for Health Research, Irunlarrea 4, 31008 Pamplona, Navarra Spain; Paediatric-cardiology Unit, Complejo Hospitalario de Navarra, IdiSNA, Navarra Institute for Health Research, Irunlarrea 4, 31008 Pamplona, Navarra Spain; Cellular Genetics Unit, Policlínica Gipuzkoa, Paseo Miramón 174, Donostia, 20014 Gipuzkoa, Spain

**Keywords:** 17q21.31 Microdeletion syndrome, Chromosomal rearrangement, Genotype-phenotype association, *KANSL1*, RASopathies

## Abstract

**Background:**

Chromosome 17q21.31 microdeletion syndrome is a multisystem genomic disorder caused by a recurrent 600-kb-long deletion, or haploinsufficiency of the chromatin modifier gene *KANSL1*, which maps to that region. Patients with *KANSL1* intragenic mutations have been reported to display the major clinical features of 17q21.31 microdeletion syndrome. However, they did not exhibit the full clinical spectrum of this disorder, which might indicate that an additional gene or genes, located in the 17q21.31 locus, might also be involved in the syndrome’s phenotype.

**Methods:**

Conventional and molecular karyotypes were performed on a female patient with intellectual disability, agenesis of the corpus callosum, heart defects, hydronephrosis, hypotonia, pigmentary skin anomalies and facial dysmorphic features. FISH analysis was conducted for chromosomal breakpoint localization. qRT-PCR was applied for the comparative gene expression of *KANSL1* gene in the patient and a control group.

**Results:**

Herein, we present the first report of disruption and haploinsufficiency of the *KANSL1* gene, secondary to a t(1;17)(q12;q21)dn chromosomal translocation in a girl that also carried a *de novo* ~289-kb deletion on 16p11.2. *KANSL1* gene expression studies and comparative clinical analysis of patients with 17q21.31 deletions and intragenic *KANSL1* gene defects indicate that *KANSL1* dysfunction is associated with the full spectrum of the 17q21.31 microdeletion syndrome, which includes characteristic facial features, hypotonia, intellectual disability, and structural defects of the brain, heart and genitourinary system, as well as, musculoskeletal and neuroectodermal anomalies. Moreover, we provide further evidence for the overlapping clinical phenotype of this condition with the cardio-facio-cutaneous (CFC) syndrome.

**Conclusions:**

*KANSL1* gene haploinsufficiency is necessary and sufficient to cause the full spectrum of the 17q21.31 microdeletion syndrome. We hypothesize that the *KANSL1* gene might have an effect on the Ras/mitogen-activated protein kinase (MAPK) pathway activity, which is known to be deregulated in the CFC syndrome. This pathway has a crucial role in the development of the heart and craniofacial morphology, as well as the skin, eye, brain and musculoskeletal systems.

## Background

The 17q21.31 microdeletion syndrome (del17q21.31), also known as the Koolen-De Vries Syndrome, is characterized by distinctive facial features, hypotonia, intellectual disability and friendly/amiable behavior [1, 2]. Over 50 % of the cases also present with structural defects of the brain (agenesis of the corpus callosum, ventricle dilatation), heart (aortic and/or pulmonary stenosis, ventricular and atrial septal defects) and/or genitourinary system. Additional signs include musculoskeletal features and hair, dental and pigmentary skin anomalies [2–6] that, in some cases, resemble those seen in the cardio-facio-cutaneous (CFC) syndrome.

The critical deleted region of the del17q21.31 syndrome was initially defined as a 424-kb segment [2], and later narrowed down to 160–274 kb containing *MAPT*, *STH* and *KANSL1* genes [5, 7]. Recent reports of small atypical deletions and heterozygous intragenic mutations in *KANSL1* demonstrated that haploinsufficiency of this gene is responsible for the major clinical signs of the syndrome [8, 9]. However, with the exception of a ventricular septal defect that spontaneously corrected, structural defects were not present in patients with intragenic *KANSL1* mutations. Therefore, it is plausible that additional genes at the 17q21.31 locus might account for the severity of the clinical phenotype of the del17q21.31 syndrome.

Analysis of apparently balanced chromosomal abnormalities associated with developmental disorders has been a successful approach to gene discovery, as the abnormal associated phenotype can be caused by hidden genomic defects at the molecular level [10, 11]. Herein, we report on the first patient to be described to have a *de novo* (1;17) translocation that truncates the *KANSL1* gene. In addition, we also reviewed the phenotype of cases with 17q21.31 microdeletions and *KANSL1* intragenic defects, in an attempt to further define the full clinical spectrum of the syndrome and understand the molecular mechanisms associated with deleterious *KANSL1* alleles.

## Patient and methods

### Patient

**Fig. 1:**
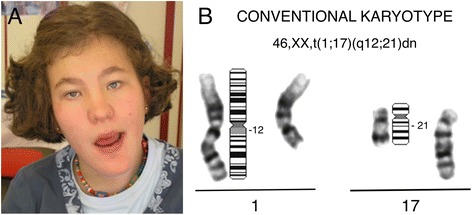
Individual with t(1;17)dn. **a** Facial features of the patient. **b** G-banding partial karyotype showing the apparently balanced translocation between chromosomes 1 and 17

The proband for our study was a girl born after a 36-week gestation complicated by fetal hydrocephaly. She weighed 1860 g, measured 45.5 cm and had a head circumference (OFC) of 30.6 cm. She presented with facial dysmorphic features, hypotonia, dilatation of cerebral ventricles, agenesis of the corpus callosum, aortic stenosis, bicuspid aorta and bilateral hydronephrosis. During the neonatal period she had severe feeding difficulties and frequent urinary infections that were caused by vesico-ureteral reflux and required surgical treatment. Her psychomotor milestones were delayed. She walked at 4 years, had language difficulties and needed special education. Facial characteristics are shown in Fig. 1a. She had a long face, short and upslanting palpebral fissures, hypertelorism, epicanthal folds, ptosis of the eyelids, iris heterochromia, broad nasal bridge, bulbous nose with thick, hypoplastic nares, long chin and absence of permanent lateral incisors. Her hair was coarse and thick. She also presented numerous nevi, *café au lait* spots and hypopigmented areas. At the age of 13 years, she showed severe thoracic kyphosis, moderate/severe intellectual disability, speech and deficit attention disorders, and a compulsive appetite that required strict diet control. Continuous cardiac and renal evaluations demonstrated mild pulmonary stenosis, peripheral cyanosis, vasomotor liability and increased size of the left ventricle, as well as a chronic renal disease with hypertension and hyperproteinuria. At her last examination (19 years of age) her weight, height and OFC were at the 90th, 10th and 50th percentiles, respectively.

The Ethics Committee at our institution (Comité Ético de Investigación Clínica de Navarra—CEIC) approved this study. Written informed consent was obtained from the patient’s parents.

### Conventional and molecular karyotype

Metaphase chromosomes were obtained from cultured peripheral lymphocytes of our patient and her parents, and a standard GTL-banding karyotype was performed for conventional cytogenetic analysis. Genomic DNA was extracted from peripheral blood cells of the patient for array-based Comparative Genomic Hybridization (aCGH) studies (Agilent Technologies). The patient was studied with a 105-K platform and a mixture of 1 μg test Cy5-labeled DNA and 1 μg male reference (Promega) Cy3-labeled DNA was used in the hybridization. The software Genomic Workbench 7.0 was used for the bioinformatic analysis of the copy number variants (CNVs). An altered copy number of DNA was considered when a region contained a minimum of five consecutive probes. Locations are based on hg18/NCBI mapview build 36.

### Fluorescence in situ hybridization (FISH)

Metaphase spreads were obtained from phytohaemagglutinin-stimulated peripheral blood lymphocytes of the patient and both parents. Bacterial artificial chromosome (BAC) clones were selected from the *Centro de Regulación Genómica* Genome Browser [12]. Plasmid DNA was extracted from clones using QIAprep Spin Miniprep Kit (Qiagen) and labeled with Spectrum Orange dUTP (SpO) or Spectrum Green dUTP (SpG) using the Nick Translation Reagent Kit (Abbott Molecular Inc.). FISH experiments were carried out according to standard procedures. The slides were examined using a Nikon Eclipse E400 with appropriate filters for Spectrum Orange, Spectrum Green and the UV Filter for the DAPI nuclear counterstain. The signals were recorded with a CCD camera and processed by ISIS v5.1 fluorescence imaging system (MetaSystems).

### Gene expression assay

Quantitative reverse-transcription polymerase chain reaction (qRT-PCR) was performed with RNA extracted from the patient and five control individuals. Total RNA was isolated from whole blood using the QIAamp RNA Blood Mini kit (Qiagen), following the manufacturer’s instructions. cDNA synthesis was performed with 1 μg of RNA using a TaqMan Reverse Transcription Reagents kit (Invitrogen-Life Technologies) in a total volume of 50 μL. Primer and probe mixtures for *KANSL1* (Hs00393805_m1, localized at exon boundary 7–8, NM_001193465.1) and the endogenous gene *GAPDH* (Hs99999905_m1, exon location 3, NM_002046.3) were supplied by Applied Biosystems. The PCR reactions were run in a 7300 Real Time PCR System (Applied Biosystems). All samples of cDNA (40 ng per well) were run in triplicate in 20 μL reaction volumes. The thermal cycling parameters were the standard conditions of the Real Time PCR System. Relative differences in transcript levels were quantified with the ΔΔCt method and data are reported as the fold-change in expression of the proband relative to the mean ± SEM of the control group.

## Results

### Balanced *de novo* translocation (1;17)

The patient’s karyotype analysis revealed the presence of an apparently balanced *de novo* translocation, designated 46,XX,t(1;17)(q12;q21)dn (Fig. 1b).

### *De novo* microdeletion on the 16p11.2 atypical/distal region

**Fig. 2:**
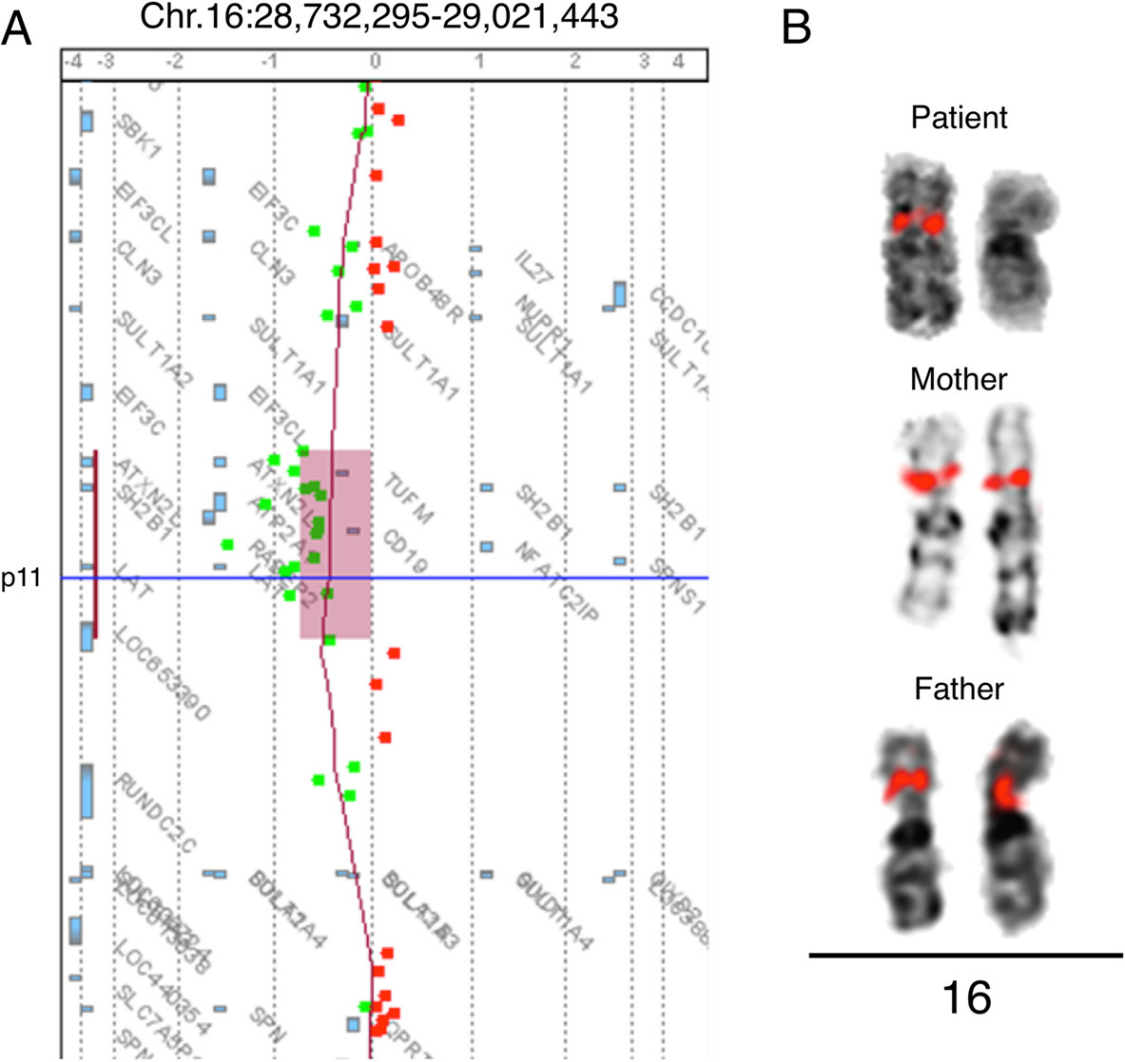
aCGH and FISH experiments on 16p11.2. **a** A detail of the patient’s aCGH analysis showing a ~289-kb deletion on the chromosome 16p11.2 distal region. **b** FISH experiments on the patient and both parents using RP11-264B17 (SpO) BAC clone. Only one signal was observed on 16p11.2 in the patient, confirming the deletion found in aCGH. Two hybridization signals were observed in each parent, indicating the *de novo* origin of the deletion. Location of RP11-264B17 probe based on hg18: 28786266–28936684

The aCGH study detected an ~289-kb deletion on the 16p11.2 atypical/distal region, flanking the recurrent microdeletion/duplication locus (Fig. 2a). No genomic imbalances were observed at translocation breakpoints or other genomic regions. Additional FISH analysis using the RP11-264B17 BAC probe confirmed the *de novo* origin of the 16p11.2 distal deletion in the patient (Fig. 2b). Combining the results of the GTL-banding, FISH and aCGH experiments, we concluded that the patient had the karyotype 46,XX,t(1;17)(q12;q21)dn.ish del(16)(q11.2q11.2)(RP11-264B17-). arr[hg18] 16q11.2(28,732,295-29,021,443) × 1 dn.

### *KANSL1* disruption and reduced gene expression

**Fig. 3:**
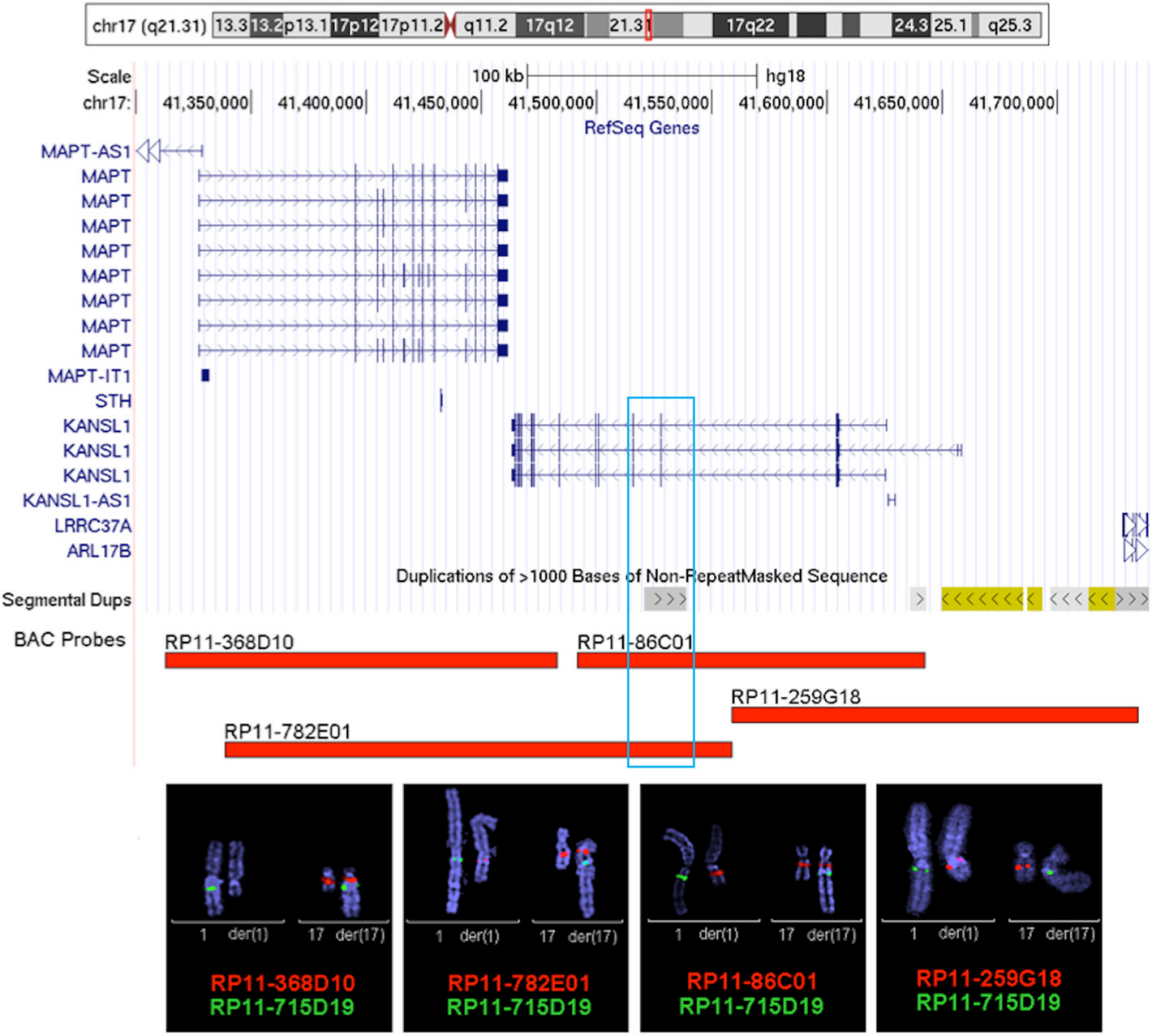
Mapping of the breakpoint on chromosome 17. Genomic context for the 17q21.31 breakpoint studied by FISH showing the genes (different transcript variants represented) and segmental duplications mapping in the region, and the position of the FISH probes RP11-368D10 (41315374–41484448), RP11-782E01 (41339059–41559177), RP11-86C01 (41493577–41644115) and RP11-259G18 (41559185–41734024). Coordinates are given according to the human reference sequence hg18 (adapted from the UCSC genome browser [20]). RP11-368D10 BAC probe gives red signal on chromosomes 17 and der(17). RP11-782E01 probe gives red signal on chromosomes 17, der(17) and der(1), overlapping the translocation breakpoint. RP11-86C01 probe gives signal on chromosomes 17, der(1) and der(17), overlapping the translocation breakpoint on *KANSL1* gene. RP11-259G18 BAC probe gives signal on chromosomes 1 and der(1). RP11-715D19 probe (SpG) has been used as a guide on chromosomes 1 and der(17). The blue box represents the putative region for the *KANSL1* gene disruption

FISH analysis, using the overlapping BAC clones RP11-782E01 and RP11-86C01 on 17q21.31, revealed hybridization signals on both derivative chromosomes (1 and 17). Additional studies with flanking probes RP11-368D10 and RP11-259G18 showed one signal on der(17) and one on der(1) chromosomes, respectively. These results indicated that RP11-782E01 and RP11-86C01 BAC probes spanned the translocation breakpoint, placing the breakpoint within a 65.6-kb region of overlap at the 17q21.31 band, which would disrupt the *KANSL1* gene (Fig. 3). This overlapping region contains a small segmental duplication, which might have contributed to a non-allelic homologous recombination event, favoring the chromosome disruption at this point.

**Fig. 4:**
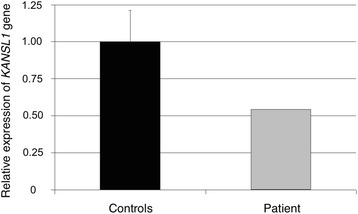
Quantification of *KANSL1* gene expression levels by qRT-PCR. The expression level of *KANSL1* gene on the patient bearing the t(1;17) (grey bar) was half that observed in the control group (black bar)

To determine how *KANSL1* gene expression was affected by the *de novo* translocation, transcript levels were assessed by qRT-PCR. The relative differences in transcript levels corresponding to *KANSL1* in the patient were half of those observed in controls, correlating with the finding of the *KANSL1* gene disruption in one chromosome (Fig. 4).

## Discussion

We present the first report of haploinsufficiency of the *KANSL1* gene caused by gene interruption, secondary to a *de novo* (1;17)(q12;q21) chromosomal translocation. The patient, who also carries an ~289-kb deletion flanking the recurrent 16p11.2 region, shows the complete phenotype of the 17q21.31 microdeletion syndrome, including characteristic facial features, hypotonia, intellectual disability, and structural defects of the brain, heart and genitourinary system, as well as musculoskeletal and neuroectodermal anomalies.

To analyze the association of the *KANSL1* gene disruption with the patient’s phenotype, we compared the frequencies of signs previously described in cases with small (424 kb) and large (502–800 kb) 17q21.31 deletions with patients carrying a *KANSL1* gene defect, including the present case. As shown in Table 1, the severity of clinical presentation of the 17q21.31 syndrome does not correlate with the size of the deletion. Moreover, our patient presented with most of the structural defects, facial features and cognitive characteristics previously described in patients with that syndrome. It could be argued, however, that her phenotype might be attributable, at least partially, to the presence of the 16p11.2 microdeletion. This deletion, which overlaps with that described in the 16p11.2 deletion syndrome (220 kb), includes nine genes (*ATXN2L*, *TUFM*, *SH2B1*, *ATP2A1*, *RABEP2*, *CD19*, *NFATC2IP*, *SPNS1* and *LAT*). Interestingly, *SH2B1* is known to be involved in leptin and insulin signaling [13] and has been reported as a predisposing factor for obesity [14]. In agreement with this hypothesis, our patient showed a compulsive appetite with difficulty in managing her weight that required strict diet control. Functions of other genes included in the 16p11.2-deleted region are currently unknown, but they do not seem to make a clear and significant contribution to the phenotype. Some authors have described cases with distal 16p11.2 deletion showing developmental delay, behavioral problems and unusual facial morphology. Although detailed clinical information may not have been available for most patients participating in large study cohorts, structural birth defects have not been previously observed associated with this genomic imbalance. Therefore, we conclude that although we can not rule out that the 289-kb microdeletion on distal 16p11.2 might contribute to the severity of the intellectual disability and/or the behavioral problems in our patient, there is currently no evidence indicating that other clinical manifestations can be attributable to the presence of this genomic imbalance. On the contrary, her facial appearance and the specific brain (agenesis of the corpus callosum), cardiac (valvulo-septal defects) and renal defects (dilation of the renal system) were very similar to those previously described in cases with 17q21.31 microdeletion syndrome.

**Table 1:** Summary of clinical signs observed in patients with del17q21.31 (classical and larger sizes), *KANSL1* mutations and *KANSL1* disruption

	17q21.31 deletion Frequency (%)		*KANSL1* mutations				*KANSL1* disruption
	Classical [2]	Large^a^	Zollino *et al.* [9]	Zollino *et al.* [9]	Koolen *et al.* [8]	Koolen *et al.* [8]	Moreno-Igoa *et al.*
	424 kb	502-810 kb	p.R606X	p.R929G fsX44	c.916C>T p.(Gln306^*^)	c.1652+1G>A	t(1;17)
Age (years) at last observation			3	14	2, 11/12	13	17
Sex	9 M/13 F	6 M/4 F	F	F	F	F	F
Growth							
Intrauterine growth retardation	27	10	+	-	-	-	-
Short stature	18	30					-
Microcephaly	5	-					-
Neurological features							
Hypotonia	96	80	+	+	+	+	+
Failure to thrive		40	+	+	+	+	+
Developmental delay/intellectual disability	100	100	+	+	+	+	+
Speech disorder		50			+	+	+
Seizures		60	-	-	-	-	+
Engaging or amiable personality	89	50	+	+	+	+	-
Behavioral disorder		30					+
Facial dysmorphic features							
Broad forehead	68	40	+	+	+		+
Long face	74	70	-	+			+
Short palpebral fissures	36	10			-	+	+
Upslanting palpebral fissures	68	70	+	+	+	+	+
Ptosis	50	-	-	-	-	-	+
Epicanthal folds	68	10	+	+	+	+	+
“Pear” shaped nose	82	40	+	+	+	+	+
Large nasal bridge		30	+	+			+
Bulbous nasal tip	95	90	+	+			+
Long philtrum		10	+	+			+
Cleft palate	9	-	-	-	-	-	-
High/narrow palate	50	10					-
Large/prominent ears	59	80	+	+			-
Broad chin	42	10	+	+	+	-	+
Ophthalmological features							
Hypermetropia	36	-			-	+	-
Strabismo	45	10			-	+	-
Iris color defects (pale/heterochromia)	45	-	-	-	-	-	+
Congenital structural defects							
Brain	38	60	-	-	-	-	+
Heart defects	27	30	-	-	-	+	+
Renal & urologic anomalies	32	50	-	-	-	-	+
Criptorchidism	78	67					
Musculoskeletal features							
Slender fingers/hands	61	20					+
Dislocation of the hip	27	30	-	+	-	+	-
Joint laxity		30	+	+	+	+	+
Pectus deformity	23	10	-	-	-	-	-
Kiphosis/Scoliosis	36	30	-	-	-	-	+
Skin, hair, teeth							
Abnormal hair texture	55	30	+	+	+	+	+
Skin pigmentary abnormalities		50	-	-	-	+	+
Hipodontia		20					+

^a^Based on description of 10 individuals with large 17q21.31 deletions [3–5, 21–23]

“+”, presence of the clinical sign; “-”, absence of the clinical sign; “blank”, not assessed; “M”, male; “F”, female

The wide range of defects affecting the skin, hair, irises and teeth present in our patient are of particular interest as they indicate a role of the *KANSL1* gene in neuroectodermal developmental processes. Additionally, these neurocutaneous signs, in conjunction with the musculoskeletal and cardiac manifestations, including the myocardiopathy, overlap with the phenotype of CFC syndrome. This disorder belongs to a clinically defined group of genetic syndromes caused by germline mutations in genes that encode for components or regulators of the Ras/MAPK pathway, generically known as RASopathies [15]. The phenotypic similarity between the 17q21.31 deletion syndrome and some RASopathies might be indicative of the possible influence of the *KANSL1* gene on the Ras-MAPK pathway activity, which has a crucial role in the development of the heart and craniofacial morphology, as well as the skin, eye, brain and musculoskeletal systems.

KANSL1 is a nuclear protein identified as a member of the non-specific lethal (NSL) complex. This histone acetyltransferase (HAT) complex also includes MOF, encoded by *KAT8*, and exerts its influence on gene expression through the acetylation of histone H4, mainly H4K16 [16]. *KANSL1* is necessary and sufficient for regulating MOF acetyltransferase activity on nucleosome H4. Moreover, it is also required for the specific acetylation of p53 on K120, which is crucial for the differential and optimal transcription activation of p53 target genes, both *in vivo* and *in vitro* [17]. *BTG2* (B-cell translocation gene 2) is an early growth response gene whose promoter contains p53-binding sites that is strongly regulated by p53 [18]. Interestingly, *BTG2* is one of the mediators of the p53-dependent inhibition of H-Ras activity, which is involved in a variety of biological processes including cell growth, development, differentiation, senescence and cell death. This gene binds H-Ras (G12V) and represses its activity by reducing its GTP loading state, which, in turn, activates the Ras/MAPK signaling cascade and causes a reduction in the expression of a large number of downstream molecules [19]. Therefore, it is plausible that dysfunction of *KANSL1* might perturb the activity of p53 transcription target genes, such as *BTG2*, producing an aberrant regulation of important downstream cascades of Ras. This mechanism could explain the overlapping phenotypic features observed in the *KANSL1* haploinsufficiency phenotype and the RASopathies. Additional clinical and experimental studies will be needed to evaluate this hypothesis and further understand the molecular mechanisms associated with deleterious *KANSL1* alleles.

## Conclusions

We present the first report of haploinsufficiency of the *KANSL1* gene caused by gene interruption, secondary to a chromosomal translocation, associated with the complete phenotype of the 17q21.31 microdeletion syndrome, including brain, cardiac and renal structural defects not previously described in patients bearing *KANSL1* point mutations. This further demonstrates that dysfunction of the *KANSL1* gene is necessary and sufficient to cause the full clinical spectrum of this syndrome. We also hypothesize that the *KANSL1* gene might have an effect on the Ras/MAPK pathway activity, which is known to be deregulated in the CFC syndrome.

## Abbreviations

aCGH: Array-based comparative genomic hybridization
BAC: Bacterial artificial chromosome
CCD: Charge-coupled device
CFC: Cardio-facio-cutaneous
CNV: Copy number variant
DAPI: 4′,6-diamidino-2-phenylindole
dUTP: Deoxyuridine triphosphate
FISH: Fluorescence in situ hybridization
GTL-banding: Giemsa/Trypsin/Leishman-banding
HAT: Histone acetyltransferase
MAPK: Mitogen-activated protein kinase
NSL: Non-specific lethal
OFC: Occipital frontal circumference
qRT-PCR: Quantitative reverse-transcription polymerase chain reaction
SEM: Standard error of the mean
SpG: Spectrum green
SpO: Spectrum orange
UV: Ultraviolet
